# Estimation of Aspect Ratio of Cellulose Nanocrystals by Viscosity Measurement: Influence of Aspect Ratio Distribution and Ionic Strength

**DOI:** 10.3390/polym11050781

**Published:** 2019-05-01

**Authors:** Qiang Wu, Xiuwen Li, Qian Li, Siqun Wang, Yan Luo

**Affiliations:** 1 School of Engineering, Zhejiang A&F University, Hangzhou 311300, China; lxwkyjy@163.com (X.L.); liqian_polymer@126.com (Q.L.); 2 Zhejiang Provincial Collaborative Innovation Center for Bamboo Resources and High-Efficiency Utilization, Hangzhou 311300, China; 3 Center for Renewable Carbon, University of Tennessee, Knoxville, TN 37996, USA; 4 College of Materials Science and Engineering, Zhejiang University of Technology, Hangzhou 310014, China; luoyan@zjut.edu.cn

**Keywords:** cellulose nano-crystal, aspect ratio, distribution, intrinsic viscosity, ionic strength

## Abstract

The influence of the cellulose nanocrystal (CNC) aspect ratio (L/d) distribution and ionic strength of different salts on the L/d estimation by viscosity measurement were investigated. The L/d distribution was controlled by mixing two CNC, with different L/d, which were prepared by acid hydrolysis from wood and bacterial cellulose. The results demonstrated that the L/d distribution did not affect the accuracy of the CNC L/d estimated by viscosity measurements using the Batchelor equation, and the calculated L/d was the number-average L/d. Moreover, monovalent (NaCl), divalent (CaCl_2_), and trivalent (AlCl_3_) salts were chosen to study the influence of ionic strength on the CNC L/d estimation by viscosity measurement. It was found that NaCl and CaCl_2_ could be added to the CNC suspension to screen the electro-viscous effect and estimate the actual physical CNC L/d by viscosity measurement, and the content of NaCl and CaCl_2_ can be predicted by the Debye–Hückel theory. However, a small amount of AlCl_3_ induced CNC aggregation and increased intrinsic viscosity and predicted L/d.

## 1. Introduction

Cellulose nanocrystals (CNC) are a rod-shaped promising nanomaterial that can be isolated from different cellulose fibers by acid hydrolysis [1,2,3,4]. Due to its biodegradability, renewability, high aspect ratio, high modulus, and negligible thermal expansion, CNC have attracted great attention from researchers and are widely used in polymer reinforcement [5,6,7,8,9], prepared for functional biomaterials in applications of tissue engineering [10,11,12], food [13,14], optical [15,16,17], and water treatment [18] among others.

The aspect ratio, which is the length divided by the diameter (L/d), is an important parameter of CNC, which determines the performances of CNC reinforced polymers, functional materials and suspensions. Generally, transmission electron microscopy (TEM) and atomic force microscopy (AFM) are the main and effective measurements used to obtain the CNC L/d. Rheology is the study of the flow and deformation of matter, morphology of matter would determine its rheological behavior. Parra-Vasquez et al. [19] and Wierenga et al. [20] used the rheology method to characterize the shape factor of carbon nanotubes and rod like silica. Recently, many studies have shown that the morphology of CNC is related to its rheological behavior in aqueous suspensions [21,22,23,24,25,26,27,28,29]. Therefore, according to the basis of the dynamics of a rod-shaped molecule, the viscosity measurement was introduced to estimate the CNC L/d, and it was found that by adding a suitable amount of NaCl into the CNC suspensions, the viscosity measurement could estimate the actual physical CNC L/d [30]. Boluk et al. [31] and Lenfant et al. [32] also found that the viscosity measurement was a simple and reliable method to characterize the CNC shape factor. Although TEM and AFM are the best methods in which to characterize the morphology of CNC, viscosity measurement has its advantages. First, it is a quick method that can estimate the L/d of CNC in 2–3 h, while the TEM or AFM method requires about 8–10 h to obtain the size of the CNC [19]; second, it is a simple method as an Ubbelohde viscometer is cheap and can be used at almost every laboratory; and third, it can estimate the average L/d of the CNC in the suspensions, while the TEM or AFM methods always need analyze more than 100–500 CNC. Therefore, viscosity measurement is suitable for fast analyzing CNC L/d during its production process.

However, CNC have been prepared by using different raw materials and different acid hydrolysis conditions, which made CNC have different L/d distributions [33,34,35,36,37]. The aspect ratio distribution may affect the accuracy of the viscosity method to estimate the CNC L/d. To our knowledge, no work has reported the influence of L/d distribution on its intrinsic viscosity and predicted L/d. Moreover, to obtain the actual physical L/d, NaCl should be added into CNC suspensions to screen the electro-viscous effect [30]. Ionic strength has a great influence on the rheological behavior of CNC suspensions due to CNC have different charge density [24,38,39]; however, most studies have focused on the monovalent salt NaCl. The influence of divalent and trivalent salts on the viscosity of CNC suspension and L/d prediction by viscosity measurements have seldom investigated. Different salts may have different influences on the viscosity of CNC suspension, and further affect the CNC aspect ratio estimation. It is meaningful to study the influence of different salt on the rheological behavior of CNC suspension. Therefore, the main objective of this study was to understand the influence of the L/d distribution of the CNC and the ionic strength of monovalent, divalent, and trivalent salts on the intrinsic viscosity of dilute CNC suspensions and the L/d estimation of the CNC by the viscosity method.

## 2. Materials and Methods

### 2.1. Materials

Soft wood pulp paper purchased from Qindao Ruili Co. Ltd., Qindao, China and bacterial cellulose purchased from Hainan Yida Food Industry Co. Ltd., Haikou, China, were used to prepare CNC with different L/d. Sulfuric acid (H_2_SO_4_, 98 wt %) was supplied by Dafang Chemical, Tianjin, China. Sodium chloride (NaCl, AR), calcium chloride (CaCl_2_, AR), and aluminum chloride (AlCl_3_, AR) were obtained from the Sinopharm Chemical Reagent Company, Shanghai, China. Dialysis membrane (MD 44) with a molecular weight cut-off of 8000–14,000 was purchased from Solarbio Science and Technology Co. Ltd., Beijing, China. Deionized water was used, and all materials were used without further purification.

### 2.2. CNC Preparation

Soft wood pulp was used to prepare CNC with a low aspect ratio. First, the soft wood pulp paper was torn and cut into powder with a knife mill (FZ102, Shanghai Ke Heng Industrial Co. Ltd., Shanghai, China). Second, the pulp cellulose powders were added into 64 wt % sulfuric acid for acid hydrolysis (weight ratio of powder and acid was 1:10, reaction temperature and time was 45 °C and 60 min, respectively), and followed by adding 10-fold water to stop the reaction. The resulting suspension was centrifuged twice at 4000 g for 5 min, then dialyzed with dialysis membranes against deionized water until reaching pH ≈ 7. Afterward, the suspension was dispersed with an ultrasonic processor (JY98-IIID Ningbo Scientz Biotechnology Co. Ltd., Ningbo, China) at an output power of 1200 W to disperse the CNC for 5 min in an ice bath, then filtered with filter paper (Whatman 541, Buckinghamshire, UK) to remove large aggregates. The concentration of the CNC suspension was characterized by drying and weighing. The soft wood CNC was named CNC-A.

Bacterial cellulose was used to prepare CNC with a high aspect ratio. Ten grams of bacterial cellulose first underwent high-speed shearing by a homogenizer, and was then mixed into sulfuric acid (65 wt %) and hydrolyzed at 70 °C for 45 min with constant mechanical stirring, followed by adding 10-fold water to stop the hydrolysis reaction. Then, the suspension was centrifuged, dialyzed, and treated by ultrasonic gradually, which were the same procedures for the soft wood CNC preparation. The bacterial cellulose CNC was named CNC-B.

Although CNC-A and CNC-B themselves are not mono-dispersed in size, the mixture of CNC-A and CNC-B would control and amplify the CNC size distribution, which made it easier for us to study the influence of the L/d distribution. To obtain CNC with different L/d distributions, different mass ratios (2/8, 5/5, and 8/2) of CNC-A and CNC-B were mixed, and the CNC mixtures were named CNC-m28, CNC-m55, and CNC-m82, respectively.

### 2.3. Transmission Electron Microscopy (TEM)

A JEM-1200EX (JEOL Ltd., Tokyo, Japan) TEM was used to observe the morphologies of CNC-A and CNC-B. A CNC aqueous suspension with 0.01 wt % was used to prepare the TEM samples. First, a drop of the suspension was deposited on a carbon-coated copper grid. Then, the excess suspension was wicked off using filter paper. Afterward, a drop of uranyl acetate solution (2 wt %) was deposited on to the grid to stain the CNC samples. Finally, the extra solution was wicked off with filter paper. The length and diameter of about 100 CNC in the TEM images were measured and analyzed with Image-Pro Plus 6.0 software (Meyer Instruments, Houston, TX, USA).

### 2.4. Zeta Potential Measurement

A ZetaPALS instrument (Brookhaven Instruments, Holtsville, NY, USA) was used to characterize the zeta potentials of the CNC suspensions. At least five measurements were performed and the data were averaged, all measurements were taken at 25 °C.

### 2.5. Viscosity Measurement

An Ubbelohde viscometer with a capillary diameter of 0.56 mm was used to measure the suspension viscosity according to the Ubbelohde method [40]. The intrinsic viscosities of the suspensions were calculated by plotting the relative viscosity versus concentration by Fedors equation [41] as follows:

(1) $$\frac{1}{2\left(\eta_{r}^{1/2}-1\right)}=\frac{1}{c\left[\eta \right]}-\frac{1}{c_{m}\left[\eta \right]}$$

where *η*_r_ is the relative viscosity; [*η*] is intrinsic viscosity; *c* is the concentration of particles in the suspension (g/mL); and *c*_m_ is the concentration at the maximum packing of particles (g/mL).

The CNC suspension used for L/d estimation should be in a dilute regime, where the hydrodynamic interactions between particles can be negligible [42].

### 2.6. Aspect Ratio Estimation

When [*η*] is calculated by the viscosity measurement, the CNC aspect ratio can be estimated by the equations presented by Batchelor [42,43].

(2) $$\left[\eta \right]=\frac{8}{45}{\left(\frac{L}{d}\right)}^{2}\frac{\varepsilon f\left(\varepsilon \right)}{\rho }$$

(3) $$\varepsilon =\frac{1}{ln\left(2L/d\right)}$$

(4) $$f\left(\varepsilon \right)=\frac{1+0.64\varepsilon }{1-1.5\varepsilon }+1.659\varepsilon^{2}$$

where *ρ* is the density of the CNC, which can be considered as 1.55 g/cm^3^. *ε* and *f*(*ε*) are the functions of aspect ratio shown in Equations (3) and (4).

## 3. Results and Discussion

### 3.1. Morphology of CNC

**Figure 1:**
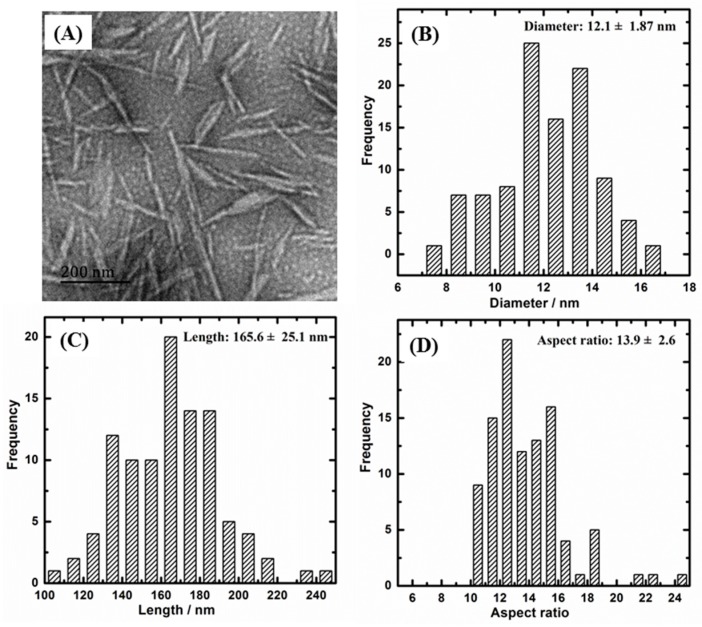
(**A**) TEM image of wood CNC (CNC-A) and its size statistics of (**B**) diameter, (**C**) length, and (**D**) aspect ratio. CNC, cellulose nanocrystal.

**Figure 2:**
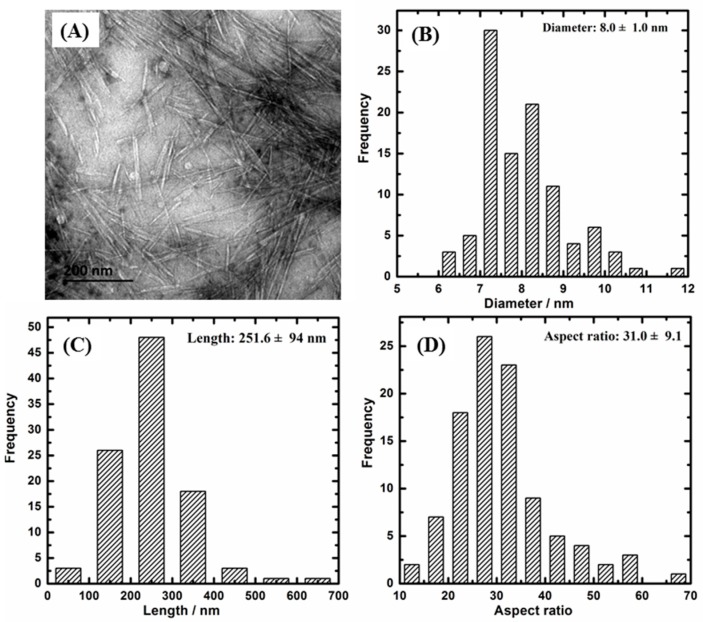
(**A**) TEM image of bacterial CNC (CNC-B) and its size statistics of (**B**) diameter, (**C**) length, and (**D**) aspect ratio.

Figure 1 and Figure 2 present the TEM images and morphology statistical results of CNC-A and CNC-B, respectively. It can be seen that both CNC-A and CNC-B were rod-like. The length and diameter of CNC-A (or CNC-B) were measured and analyzed, respectively. It was found that CNC-A was 12.1 ± 1.9 nm in diameter, 165 ± 25 nm in length, and 13.9 ± 2.6 in L/d, while CNC-B was 8.0 ± 1.0 nm in diameter, 252 ± 94 nm in length, and 31.9 ± 9.1 in L/d. CNC-B obtained from bacterial cellulose was larger in length, thinner in diameter, and higher in L/d than those of CNC-A, which was prepared with soft wood. Moreover, the zeta potential of CNC-A and CNC-B were −42.5 and −38.0 mV, respectively, which demonstrated that both CNC were stable in suspension.

### 3.2. CNC Aspect Ratio Prediction by Viscosity Measurement

In order to obtain the actual physical L/d by the viscosity method, NaCl was added into the CNC suspension to screen the electro-viscous effect [30,31,44]. Additionally, the NaCl concentration could be estimated by Debye–Hückel theory where the Debye length (*κ*^−1^) is equal to the CNC diameter [30]. The Debye–Hückel theory is presented below.

(5) $$\kappa^{-1}=\sqrt{\frac{\varepsilon_{r}\varepsilon_{0}k_{B}T}{e^{2}N_{A}\sum z_{i}^{2}n_{i,\infty }}}$$

where *ε*_r_ is the relative permittivity; *ε_0_* is the vacuum permittivity; *k*_B_ is the Boltzmann constant; *T* is the Kelvin temperature; *z*_i_ is the valence of the solvated ions; *n*_i,∞_ is their concentration (mol/m^3^); *N*_A_ is Avogadro’s number; and *e* is the net electron charge. The diameters of CNC-A and CNC-B were 12 and 8 nm, respectively. According to the Debye–Hückel theory, if *κ*^−1^ = 10 nm, then the NaCl concentration is about 1.0 mM. Therefore, 1.0 mM NaCl was added into both the CNC-A and CNC-B suspensions to predict the actual physical L/d. 

**Figure 3:**
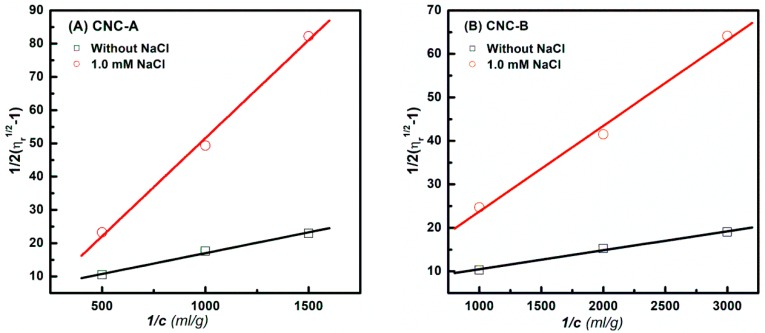
Fedors plots for (**A**) CNC-A and (**B**) CNC-B suspensions with and without 1.0 mM NaCl.

For rod-like particles, the volume fraction (*φ*) of the CNC suspension for the viscosity measurement should be *φ* < (*d*/*L*)^2^. Therefore, according to the TEM results, the volume fraction used for the viscosity measurement in this study was 0.001 for all CNC mixtures. Figure 3 shows the relationship between $\frac{1}{2\left(\eta_{r}^{1/2}-1\right)}$ and 1/*c* for the CNC-A and CNC-B suspensions with and without 1.0 mM NaCl. The Fedors equation was used to calculate the [*η*], then the CNC L/d was estimated by the Batchelor equation. The [*η*] and L/d are listed in Table 1. It was found that adding NaCl screened the electrostatic effect of the CNC, and both CNC-A and CNC-B showed smaller [*η*] and calculated L/d. Moreover, the calculated L/d of CNC-A (14) and CNC-B (30) were very close to the TEM statistical results of CNC-A (13.9 ± 2.6) and CNC-B (31.9 ± 9.1), confirming that the adding 1.0 mM NaCl to the CNC-A and CNC-B suspensions could accurately predict the actual physical L/d of CNC-A and CNC-B.

### 3.3. Influence of Aspect Ratio Distribution

In order to study the influence of the L/d distribution of the CNC on the intrinsic viscosity and predicted L/d, different mass ratios (2/8, 5/5, and 8/2) of CNC-A and CNC-B were mixed. According to the morphology, statistical results of CNC-A and CNC-B, the number ratio, mass-average L/d, and number-average L/d were calculated by using the equations below and are listed in Table 2.

(6) n1n2=m1L1d12m2L2d22

(7) $$P_{m}=\frac{m_{1}}{m_{1}+m_{2}}P_{1}+\frac{m_{2}}{m_{1}+w_{2}}P_{2}$$

(8) $$P_{n}=\frac{n_{1}}{n_{1}+n_{2}}P_{1}+\frac{n_{1}}{n_{1}+n_{2}}P_{2}$$

where *m*_1_*/m*_2_ and *n*_1_*/n*_2_ are the mass ratio and number ratio of CNC-A to CNC-B in mixture; *P*_m_ and *P*_n_ are the mass average and number average L/d of the CNC mixtures; *L*_1_ (*L*_2_), *d*_1_ (*d*_2_), and *P*_1_(*P*_2_) are the length, diameter and L/d of CNC-A (CNC-B) measured with TEM. 

**Figure 4:**
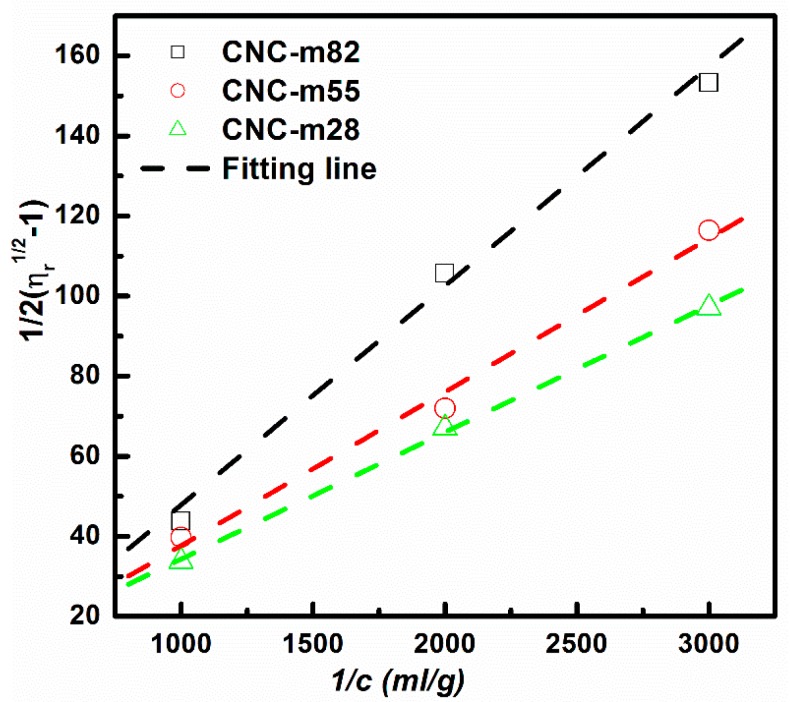
Fedors plots for CNC-A and CNC-B mixed suspensions with 1.0 mM NaCl.

As 1.0 mM NaCl can screen the electro-viscous effect of CNC-A and CNC-B, and predict the actual physical L/d. A quantity of 1.0 mM NaCl was also added to the CNC mixed suspensions to predict the L/d of CNC-m28, CNC-m55, and CNC-m82. Figure 4 presents the relationship between $\frac{1}{2\left(\eta_{r}^{1/2}-1\right)}$ and 1/*c* for the mixed CNC suspensions with 1.0 mM NaCl. The [*η*] and L/d were calculated and listed in Table 2.

It was found that the L/d predicted by the intrinsic viscosity of CNC-m28, CNC-m55, and CNC-m82 were 30, 28, and 18, respectively, which were basically equal to the number-average L/d calculated by TEM statistical data. Therefore, it was demonstrated that the CNC L/d distribution did not affect the accuracy of the CNC L/d estimated by the viscosity method. Furthermore, the calculated aspect ratio was the number-average L/d.

### 3.4. Influence of Ionic Strength

**Figure 5:**
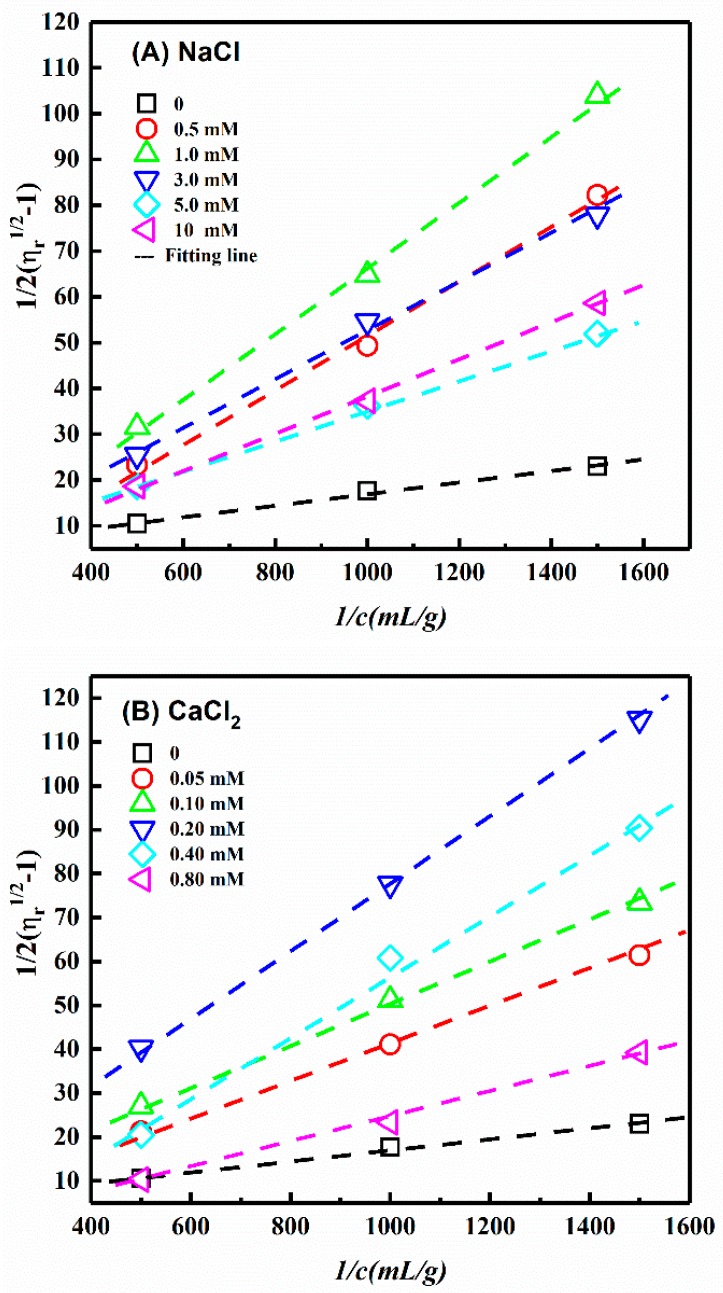

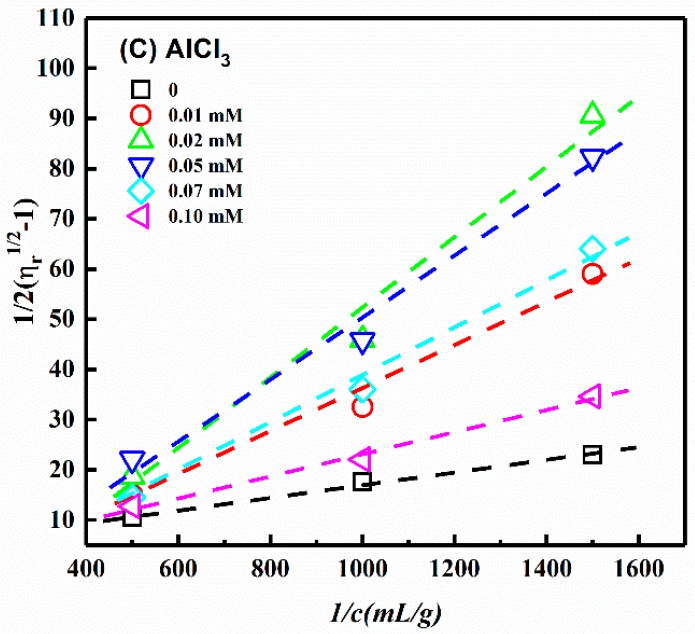
Fedors plots for CNC-A suspensions with different concentration of (**A**) NaCl, (**B**) CaCl_2_, and (**C**) AlCl_3_.

In this section, monovalent (NaCl), divalent (CaCl_2_), and trivalent (AlCl_3_) salts were added into CNC-A suspensions to investigate the influence of ionic strength. Figure 5 presents the Fedors plots for the CNC-A suspensions with different concentrations of NaCl, CaCl_2_, and AlCl_3_. The [*η*] and L/d were calculated and are listed in Table 3. For all of the CNC-A suspensions with different salts, the [*η*] and predicted L/d initially decreased with increasing salt concentration, as the addition of salts screened the electro-viscous effect. Then, the [*η*] and predicted L/d increased due to CNC aggregation (flocculation) at higher salt concentrations. The minimum calculated aspect ratios of CNC-A were 14, obtained by adding 1.0 mM NaCl, 0.2 mM CaCl_2_, and 0.02–0.05 mM AlCl_3_ in CNC-A suspension, respectively. The minimum calculated L/d of CNC-A was equivalent to the TEM analyzed data (13.9 ± 2.6).

According to the Debye–Hückel theory, *κ*^−1^ = *d* (*d* = 12.1 nm) was used to predict the amount of salt to be added [30], hence, the NaCl, CaCl_2_, and AlCl_3_ concentration were 0.6, 0.2, and 0.1 mM, respectively. It was found that the NaCl and CaCl_2_ concentration, added into CNC-A suspension, to estimate the actual physical L/d were close to the Debye–Hückel theory predicting the salt concentration. The NaCl concentration range was from 0.5 to 3.0 mM (0.6 mM predicted), and the CaCl_2_ concentration range was from 0.2 to 0.4 mM (0.2 mM predicted). However, the AlCl_3_ concentration (0.02–0.05 mM) added into the CNC-A suspension to estimate the actual physical CNC-A L/d, was much lower than the concentration (0.1 mM) predicted by the Debye–Hückel theory. This can be attributed to Al^3+^ acting as a physical cross-linking agent for the CNC with negative charges. Masruchin et al. [45] and Dong et al. [46] also found that divalent or trivalent cations could induce a nano-cellulose suspension to form hydrogels.

Because CNC has a diameter distribution, the corresponding adding salt should be in a range. Let us take NaCl as an example, the diameter of CNC-A is 12.1 ± 1.9 nm (10.2–14 nm), therefore, the NaCl concentration range can be calculated by Debye–Hückel theory using *κ*^−1^ = 10.2 and 14 nm. The calculated NaCl range was between 0.5–1 mM, which was close to the experiment results (0.5–3.0 mM). The large experimental NaCl range may be attributed to the difference between screen effect and aggregation. Furthermore, for the accurate estimation of the actual physical CNC L/d, the NaCl concentration range (0.5–3.0 mM) added into the CNC-A suspension was much wider than that of CaCl_2_ (0.2–0.4 mM). Therefore, NaCl should be the optimal choice to add into CNC suspensions to estimate the actual physical L/d.

## 4. Conclusions

Two CNC with different aspect ratios were mixed to study the aspect ratio distributions on the aspect ratio predication by viscosity measurement. The results showed that the L/d distribution did not affect the accuracy of the L/d predicted by the viscosity method, and the calculated aspect ratio was a number-average L/d, which indicates that the viscosity method is a reliable method to characterize the CNC aspect ratio. Moreover, it was found that the Debye–Hückel theory could predict the NaCl and CaCl_2_ concentrations to estimate the actual physical CNC aspect ratio. However, the AlCl_3_ concentration could not be predicted by the Debye–Hückel theory, as Al^3+^ acted as a physical cross-linking agent for CNC with negative charges. Finally, we suggest that NaCl should be the optimal choice to add into CNC suspensions to estimate their actual physical L/d.

## Figures and Tables

**Table 1:** Intrinsic viscosities and calculated L/d of CNC-A and CNC-B without and with 1.0 mM NaCl.

NaCl Concentration (mM)	CNC-A		CNC-B	
	[*η*] (mL/g)	L/d	[*η*] (mL/g)	L/d
0	120	40	344	73
1.0	21	14	76	30

**Table 2:** Mass ratio, number ratio, number average L/d, mass average L/d, intrinsic viscosity, and predicted L/d of CNC-m28, CNC-m55, and CNC-m82.

Samples	*m*_1_/*m*_2_	*n*_1_/*n*_2_	*P*_m_	*P*_n_	[*η*] (mL/g) (Experiment)	L/d (Experiment)
CNC-m28	2:8	0.166:1	28.3	29.3	75	30
CNC-m55	5:5	0.664:1	22.9	24.7	55	28
CNC-m82	8:2	2.66:1	17.5	18.8	33	18

**Table 3:** Intrinsic viscosities of CNC-A suspensions and calculated aspect ratio of CNC with various NaCl, CaCl_2_, and AlCl_3_ concentrations.

NaCl Concentration (mM)	[*η*] (mL/g)	L/d	CaCl_2_ Concentration (mM)	[*η*] (mL/g)	L/d	AlCl_3_ Concentration (mM)	[*η*] (mL/g)	L/d
0	120	40	0	120	40	0	120	40
0.5	25	16	0.05	37	20	0.01	34	19
1.0	21	14	0.1	32	18	0.02	21	14
3.0	29	17	0.2	20	14	0.05	22	14
5.0	40	21	0.4	26	16	0.07	24	18
10.0	45	23	0.8	52	24	0.10	68	29
